# The *stn1*-*sz2* Mutant Provides New Insight into the Impacts of Telomeric Cdc13-Stn1-Ten1 Dysfunction on Cell Cycle Progression

**DOI:** 10.3390/cells14110784

**Published:** 2025-05-26

**Authors:** Nathalie Grandin, Michel Charbonneau

**Affiliations:** GReD Institute, CNRS UMR6293, INSERM U1103, Faculty of Medicine, University Clermont-Auvergne, 28 Place Henri Dunant, BP 38, 63001 Clermont-Ferrand Cedex, France

**Keywords:** budding yeast telomeres, Cdc13-Stn1-Ten1 complex, cell cycle, Bub2 and Mad2 spindle checkpoints, Mec1 DNA damage checkpoint

## Abstract

The conserved and essential Cdc13/CTC1-Stn1-Ten1 telomeric complex (CST) ensures chromosome stability by protecting telomere ends and regulating telomerase accessibility. In a recent study, we uncovered mutants of the *S*. *cerevisiae* CST, in which damage was sensed by the two major G2/M spindle checkpoints (one is Bub2-dependent and the other one Mad2-dependent), as well as the major G2/M DNA damage checkpoint (Mec1-dependent). In this study, we found, by fluorescence microscopy, that the stability of the mitotic tubulin spindle was profoundly affected in the best-studied of these mutants, *stn1*-*sz2*. Additional data from genetic analyses suggested the potential involvement of Stu1 and Stu2, as well as Slk19, in these defects. Throughout this study, we compared the phenotypes of *stn1*-*sz2* with those of *cdc13*-*1*, the best-studied CST mutant, which also serves as a prototype of telomere-damage-characterized CST mutants. We propose that *stn1*-*sz2* represents the prototype of *cst* mutants characterized by tubulin spindle damage. These newly described phenotypes potentially represent the basis for identifying new functions of the CST telomeric complex. These functions might consist of ensuring correct chromosome segregation through the stabilization of the mitotic spindle.

## 1. Introduction

Telomeres, the ends of linear chromosomes, play a major role in ensuring the overall stability of the entire genome. Indeed, telomeres form a specific protection cap, made of TG-rich DNA sequences and specialized binding proteins, that prevents them from being recognized as DNA double-strand breaks by DNA damage-signaling and repair machineries [1]. When the telomeric cap fails to fully protect the ends of the chromosomes, they progressively degrade from the telomere side to the centromere side, leading to inappropriate recombination between the exposed telomeric sequences, end-to-end fusion, and cell death as soon as essential genes become consumed by chromosome degradation. The loss of telomere end protection was first described in mutants of *S*. *cerevisiae* Cdc13, a subunit of the Cdc13-Stn1-Ten1 (CST) complex [2].

It is important to note that telomeres represent fragile structures even in the absence of any accidental damage. This is because of the intrinsic property of DNA synthesis that leads to the incomplete replication of the lagging strand [3]. This so-called end replication problem, together with oxidative damage and exonucleolytic processes, provokes irremediable telomere shortening in dividing cells. Once telomere erosion has reached a certain threshold, telomeres lose their attached proteins, triggering a signal of damage that is sensed by cell cycle checkpoints, leading to senescence or apoptosis. In dividing cells, telomerase, a specialized reverse transcriptase [4], and CST compensate for the erosion of telomeres resulting from the end replication problem [3,5]. However, since the cells of most human tissues do not actively divide, they do not need the action of telomerase to re-elongate their eroding telomeres. In fact, telomerase is naturally inactivated (via transcriptional regulation) in most human tissues, apart from germinal and stem cells. This most likely represents a mechanism of protection, through which aging cells with accumulated damage can no longer continue to divide. If telomerase reactivation occurred in these cells despite this mechanism, there would certainly be increases in the occurrence and propagation of genetic abnormalities, hence the importance of telomeres and telomerase in cancer. Cells with telomeres left unprotected are more likely to become cancerous and immortal when, together with the simultaneous inactivation of a cell cycle checkpoint, telomerase or an alternative lengthening of the telomere pathway (ALT) is inappropriately reactivated [6,7].

**Figure 1:**
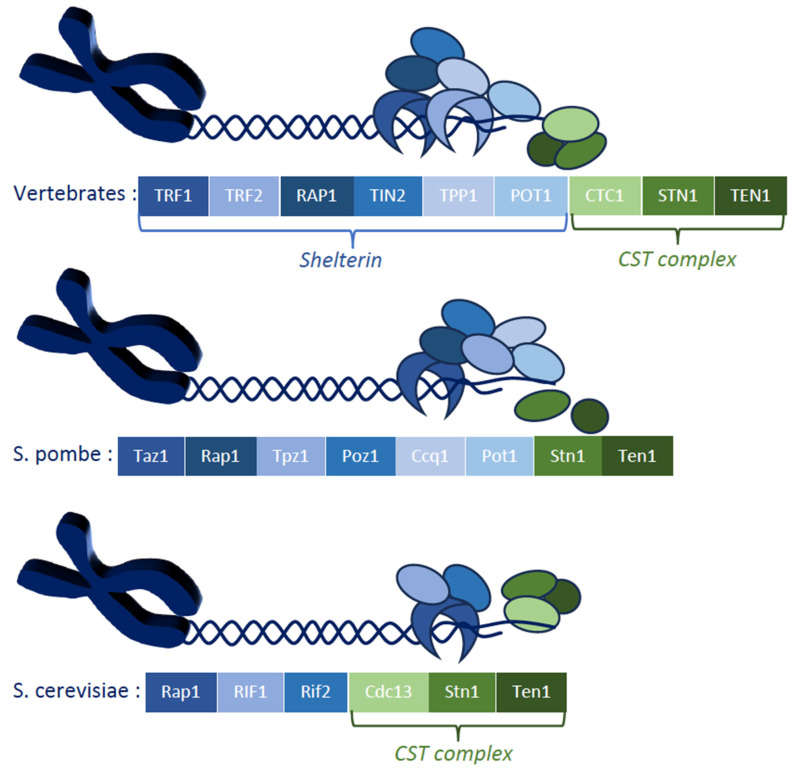
Simplified molecular architecture of chromosome-end protection and telomerase recruitment provided by the shelterin and CST complexes in three model organisms. The vertebrate telomere protection system comprises the shelterin complex, which consists of six proteins, TRF1, TRF2, RAP1, TIN2, TPP1, and POT1, and the CST complex, composed of CTC1, STN1, and TEN1. The *Schizosaccharomyces pombe* yeast shelterin complex is similar to that in vertebrates, comprising the Taz1, Rap1, Tpz1, Poz1, Ccq1, and Pot1 subunits, with globally similar functions to those in vertebrates. On the other hand, the *Saccharomyces cerevisiae* yeast possesses a CST complex, but it does not have a shelterin complex, although double-stranded telomeric DNA-binding proteins, such as Rap1, Rif1, and Rif2, assume some of shelterin’s roles.

Alongside telomerase, the two major chromosome-end protection protein complexes are shelterin and CST (Figure 1). The first CST (Cdc13-Stn1-Ten1) complex identified was found in the budding yeast *Saccharomyces cerevisiae*, in which it plays a central role in telomere homeostasis and protection through telomere capping, as well as in telomerase activation and inhibition [2,8,9,10]. Initially, the CST complex was thought to be specific to *S*. *cerevisiae* and absent in vertebrates, mainly due to the previous identification of shelterin, a complex of six proteins (TRF1, TRF2, RAP1, TIN2, TPP1, and POT1) that have complementary roles in telomere protection and telomere length regulation [11,12,13]. However, strikingly, orthologs of the three *S*. *cerevisiae* CST proteins were discovered a few years later in humans, mice, and even plants [14,15]. In the fission yeast *Schizosaccharomyces pombe*, another well-known model system for telomere biology and the cell cycle, Stn1 and Ten1, but not Cdc13/CTC1, were identified as CST subunits [16] (Figure 1). In humans, CST also assumes important telomeric functions, as it limits telomerase access to telomeres [17] and associates with shieldin at damaged telomeres to regulate, in association with Polα, the fill-in of the resected overhangs to facilitate DNA repair [18]. Even more recently, in humans, the phosphorylation of the shelterin subunit POT1 was found to trigger the recruitment of CST (and the Polα/primase complex to which it is attached), thereby keeping it in an inactive state until telomerase had extended the ends of the telomeres [19].

Dysfunctions in several telomeric pathways have been implicated in cancer, aging, and other diseases, and have established the importance of the field of telomere biology research over the last three decades [20,21,22]. The two major telomeric protein complexes, shelterin and CST, together with telomerase itself, play a major role in preventing these telomeropathies [12,13,23]. Concerning CST, mutations in human CTC1 and STN1 have been found to be mainly associated with *dyskeratosis congenita* and Coats Plus ([24] and references therein), as well as several types of cancer [25,26]. In all the eukaryotic organisms in which it is present, shelterin is considered the chief guardian of the chromosome ends [11,13]. Shelterin provides chromosome end protection, but it also controls the activity of telomerase at the telomere and serves as a sensor of telomere length. Mammalian CST (just like budding yeast CST) also participates in the replication and maintenance of the telomeres [10,12,13].

In *S*. *cerevisiae*, all three CST subunits are essential for telomere end protection and are also involved in telomere length regulation. Cdc13 plays a major role in telomerase recruitment to telomere ends and in telomere end protection by capping. Stn1 also plays a specific role, being responsible, in association with DNA Polα, for the refilling of the strand previously elongated by telomerase [10]. Ten1, on the other hand, has no known telomeric-specific function. The temperature-sensitive *cdc13*-*1* mutation was the first mutation discovered in CST to cause extensive telomere degradation, producing extended single-stranded telomeric DNA that activated the Mec1-Rad9 DNA damage checkpoint [2,27]. Many *S*. *cerevisiae* mutants carrying the conditional *stn1* or *ten1* alleles have since been shown to exhibit telomere degradation and DNA damage checkpoint-mediated cell cycle arrest.

Our recent work has started to describe new *S*. *cerevisiae* mutants that simultaneously exhibit defects that are sensed by the DNA damage checkpoints and those sensed by the two major spindle checkpoints [28]. Here, we demonstrate that in one of these new CST mutants, *stn1*-*sz2*, the mitotic spindle of tubulin is profoundly destabilized, with dramatic consequences for the dynamics of the spindle pole bodies (centrosomes) and kinetochores during mitosis. Therefore, the CST complex might not only provide telomere-end protection and engage in telomere length regulation via its actions with respect to telomerase, but also ensure full stability of the mitotic spindle during events occurring prior to the segregation of the sister chromatids in anaphase. Throughout this study, *stn1*-*sz2*′s phenotypes were compared to those in the *cdc13*-*1* mutant, which is the prototype of telomeric-DNA-damage-characterized mutants of CST [2]. We propose considering *stn1*-*sz2* to be the prototype of spindle-damage-characterized mutants of CST. These newly described phenotypes concerning the mitotic spindle potentially represent the basis for identifying new functions of the CST telomeric complex.

## 2. Materials and Methods

### 2.1. Yeast Strains and Media

The *Saccharomyces cerevisiae* yeast strains used in this study were derivatives of BF264-15Daub (*ade1 his2 leu2*-*3*,*112 trp1*-*1a ura3*Δ*ns*), described previously [8]. Yeast cultures were grown at the indicated temperatures in YEP (1% yeast extract, 2% bacto-peptone, 0.005% adenine, and 0.005% uracile) supplemented with 2% glucose (YEPD) or in selective minimal media. All strains were made isogenic by back-crossing them at least five times against our genetic background. The GFP ORF (open reading frame) sequences were attached in frame to the 3′ end of the *SLK19* or *RFA1* genes cloned into one of the integrative expression vectors of the *S*. *cerevisiae* YIplac series [29]. For the construction of YIp211-*SLK19*-GFP, the last 1.26 kb sequences of the *SLK19* ORF (the total ORF is 2.46 kb long) were used to clone SalI-BamHI into Yip211 (*URA3*), and the GFP ORF sequences were inserted at the 3′ end of *SLK19* and in frame with it, between the NotI site (introduced just upstream of the BamHI site at the 3′ end of *SLK19*) and the BamHI site. The YIp211-*SLK19*-GFP plasmid was then linearized by cutting either at the unique BglII site of the *SLK19* ORF or at its unique ClaI site. The linearized plasmids were then used for integrative transformation at the *SLK19* genomic locus. The actual expression of *SLK19*-GFP in the transformed yeast strains was then analyzed by Western blotting (using an anti-GFP monoclonal antibody) and immunofluorescence microscopy. For the construction of YIp211-*RFA1*-GFP, the last 1.00 kb of the *RFA1* ORF (the total ORF is 1.86 kb long) were used to clone PstI-BamHI into Yip211 (*URA3*), and the GFP ORF sequences were inserted at the 3′ end of *RFA1* and in reading frame with it, between the NotI site (introduced just upstream of the BamHI site at the 3′ end of *RFA1*) and the BamHI sites. The YIp211-*RFA1*-GFP plasmid was then linearized by cutting either at the unique NheI site of the *RFA1* ORF or at its unique BclI site. The linearized plasmids were then used for integrative transformation at the *RFA1* genomic locus. The actual expression of *RFA1*-GFP in transformed yeast strains was then analyzed as previously described for *SLK19*-GFP. Cell viability was determined by performing the so-called “drop tests” or “spot assays”. To perform these tests, cells from exponential growth cultures were counted with a hematocytometer, and the cultures were then serially diluted by 1/10th, spotted onto YEPD (or selective medium) plates, and incubated at the desired temperatures for 2–3 days before being photographed.

### 2.2. Fluorescence and Confocal Microscopy

Cells were observed using a Zeiss (Oberkochen, Germany) LSM 800 Airyscan confocal microscope. Cells were mounted on slides in the “Vectashield with DAPI” mounting medium (Vector Laboratories, Inc; Newark, CA 94560, USA). For GFP-Tub1 visualization, cells were fixed with 37% formaldehyde for 10 min.

### 2.3. Statistical Analyses

A Student’s *t*-test conducted using Prism software (version 7.0d) was used throughout the statistical analyses. Significance numbers are given as *p*-values: ns stands for “non-significant”, * stands for a *p*-value < 0.05, ** stands for a *p*-value < 0.01, *** denotes a *p*-value < 0.001, and **** indicates a *p*-value < 0.0001.

## 3. Results

### 3.1. Checkpoint Damage Sensors Activated in the stn1-sz2 Mutant

We recently isolated mutants of the *S*. *cerevisiae* telomeric Cdc13-Stn1-Ten1 complex (CST) that had the ability to simultaneously activate the two major spindle checkpoints together with the DNA damage checkpoint, the latter being the event currently encountered in classical mutants of CST [28]. We concentrated on the already-best-documented mutant in our previous study, *stn1*-*sz2*, so now our objective was to elucidate the origins of damage and the nature of the checkpoint sensors in this mutant.

**Figure 2:**
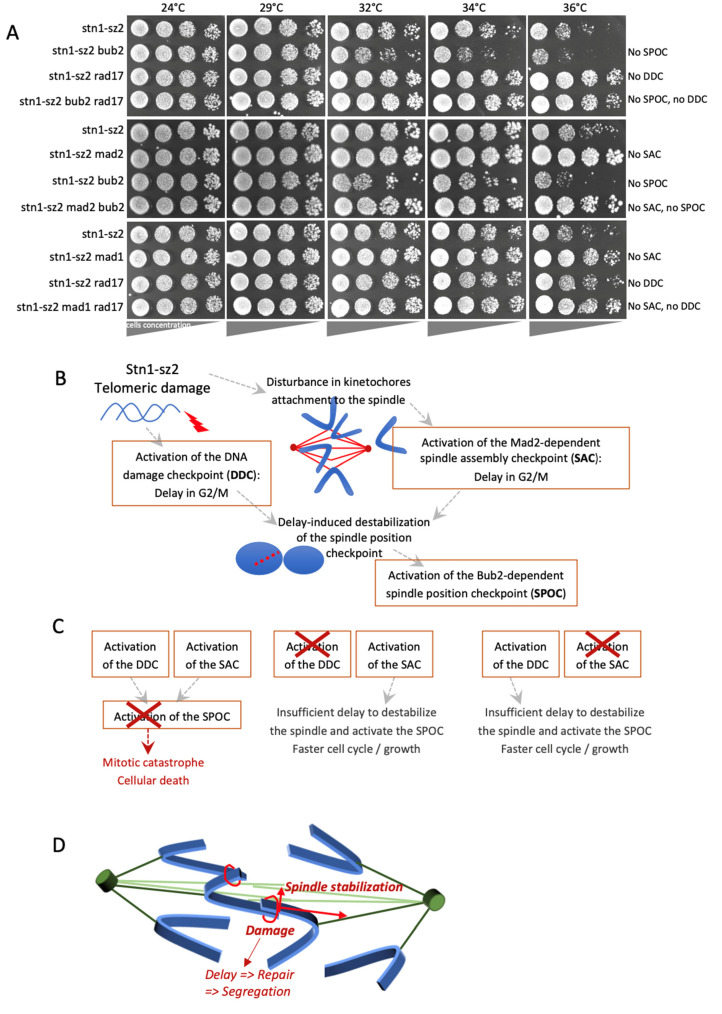
Detection of *stn1*-*sz2* defects by the three major G2/M checkpoints, namely, the spindle assembly checkpoint (SAC), the spindle orientation checkpoint (SPOC), and the Mec1-dependent DNA damage checkpoint (DDC). (**A**) The effects of the *bub2*Δ mutation (which inactivates the SPOC), *mad1*Δ or *mad2*Δ mutations (which inactivate the SAC), and *rad17*Δ mutation (which inactivates the DDC) on the growth properties of the *stn1*-*sz2* temperature-sensitive mutant cells, as indicated (see text for explanations). “Spot assays” (or “drop tests”) were performed using serial tenfold dilutions (shown from left to right) of liquid cultures grown for 3 days on YEPD plates at the indicated temperatures. (**B**) Schematic representation of the DDC and SAC events triggered by *stn1*-*sz2* damage, and an interpretation of the effects of the induced cell cycle delay on the activation of the SPOC. Refer to the main text for detailed explanations. (**C**) Schematic representation of the consequences, for checkpoint activation and growth characteristics, of inactivating either one of the three G2/M checkpoints. Refer to the main text for explanations and the growth assays shown above (in (**A**)). (**D**) Working model based on the data presented above in (**A**–**C**). Kinetochore microtubules and interpolar microtubules are presented in light green and dark green, respectively. Chromosomes are presented in blue, and spindle polar bodies are given as dark-green cylinders.

All three major G2/M checkpoints, namely, the spindle assembly checkpoint [30], the spindle orientation checkpoint [31], and the Mec1-dependent DNA damage checkpoint [32], played a significant role in the detection of *stn1*-*sz2* defects [28] (Figure 2A and Figure S1A for the quantification of the extent of growth). Interestingly, we observed two different and quite opposite effects of these checkpoint mutations on *stn1*-*sz2* growth. Thus, the inactivation of *RAD17*, resulting in DDC (“DNA Damage Checkpoint”) inhibition, or either *MAD2* or *MAD1*, leading to SAC (“Spindle Assembly Checkpoint”) inhibition, in the *stn1*-*sz2* mutant at 34–36 °C, resulted in an increase in cell viability, reminiscent of that described for the *cdc13*-*1 rad17*Δ mutant, characteristic of forcing the commencement of checkpoint-mediated cell cycle arrest [27,33,34]. On the other hand, the *stn1*-*sz2 bub2*Δ double mutant, in which *bub2*Δ inactivates the SPOC (“Spindle Orientation Checkpoint”), more classically exhibited a strong synthetic lethality that killed the cells (Figure 2A).

It is rare for a particular form of damage to simultaneously activate distinct checkpoints, as observed here. A noticeable exception in this regard, however, is nocodazole, a microtubule-depolymerizing drug, which has been shown to activate both of these G2/M spindle checkpoints [35,36], the SAC and the SPOC, involving two independent and partially redundant mechanisms [37]. To better understand the implications of the three G2/M checkpoints in the *stn1*-*sz2* mutant, we analyzed *stn1*-*sz2* mutants harboring two of these three checkpoint mutations (Figure 2A). Strikingly, the strong synthetic lethality affecting the *stn1*-*sz2 bub2*Δ double mutant was ameliorated when either *MAD1* or *RAD17* was deleted. This finding suggests that in the *stn1*-*sz2 bub2*Δ double mutant, the delay in cell cycle progression induced by the SAC or the DDC provoked some damage that was further recognized by the SPOC (Figure 2B). Presumably, the DDC and the SAC (both active in the *stn1*-*sz2 bub2*Δ mutant) individually arrested cells at a stage where additional damaged structures were recognized by the SPOC. If, at this point, either the SAC or the DDC was inactivated (*stn1*-*sz2 bub2*Δ *mad2*Δ or *stn1*-*sz2 bub2*Δ *rad17*Δ double mutant), then the arrest normally recognized by the SPOC and resulting in death no longer occurred (Figure 2C). In other words, the damaged spindle structure that is normally recognized by the SPOC in *MAD2*^+^ *RAD17*^+^ cells was no longer present in *mad2*Δ or *rad17*Δ cells because the cell cycle had progressed beyond the problematic point. These data suggest that the SAC and the SPOC might not be activated simultaneously, with the SPOC being activated only if both the DDC and the SAC were also activated. We propose that, upon the initiation of *stn1*-*sz2* damage, the additional cell cycle delays induced by the activated DDC and SAC represent a longer period during which the mitotic spindle must be stabilized in the face of telomeric damage (Figure 2B). Under these conditions, if either the DDC or SAC is inactivated, the cell cycle delay shortens, and spindle stability increases because it only has to be ensured for a shorter period (Figure 2C).

Based on these observations, it appears that CST might play a role in stabilizing the spindle. As illustrated in Figure 2D, in metaphase, it is possible that damaged telomeres tend to keep close to each other in order to attempt some sort of repair (via homologous recombination, for instance). In the next anaphase, centromeres may be pulled toward the two spindle pole bodies ((SPBs) homologs of the centrosomes). Under these conditions, the mitotic spindle must be stabilized until the telomeres are free of damage and can therefore be correctly segregated. The CST complex might be involved in this stabilization process. In the *stn1*-*sz2* mutant, specifically, spindle stabilization would not be efficient, which would activate the SAC and the SPOC.

In summary, CST is known to principally play a role in protecting the ends of telomeres against degradation by exonucleases or other DNA repair enzymes. If the telomeres are improperly protected, high levels of single-stranded telomeric DNA are generated, constituting the signal of damage sensed by the DDC. This scheme applies for classical mutants such as *cdc13*-*1* and *stn1*-*13*, in which the damage mostly activates the DDC (Figure S1B). In contrast, the *stn1*-*sz2* mutant not only activates the DDC but also, significantly, the SAC and the SPOC [28] (Figure 2A and Figure S1A). It is important to note that, in all the subsequent experiments, we used (in addition to the wild type) the *cdc13*-*1* mutant as a control because, among all the previously described mutants, it represents a model system for studying the activation of the major Mec1-dependent DNA damage checkpoint by telomeric DNA damage [2,38,39].

### 3.2. The Organization of the Mitotic Spindle Is Affected in the stn1-sz2 Mutant

Next, we used fluorescence microscopy to further test our assumption (based on the activation of the spindle checkpoints) that the stability of the mitotic spindle might be affected in the *stn1*-*sz2* mutant. Using strains expressing GFP-*TUB1* from the *TUB1* genomic locus [40], we could establish that in the *snt1*-*sz2* mutant, GFP-Tub1 signals were much weaker, and the mitotic spindles were thinner than in the wild type (Figure S2A). The ill-expression of GFP-Tub1 in the *stn1*-*sz2* mutant cells was not responsible for these defects (Figure S2B). This reduction in GFP-Tub1 signals could be due to a reduction in the nuclear accumulation of GFP-Tub1 in the *stn1*-*sz2* mutant. However, it is also possible that mitotic spindle stability is impaired in this mutant. Unfortunately, it was not possible to quantify GFP-Tub1 signals under these conditions due to the very high spread of signals in the *stn1*-*sz2* mutant (Figure S2A). However, we were able to quantify these signals when the cells had been fixed with formaldehyde, as shown below.

While preparing to visualize GFP-Tub1 labeling, we observed that a very high percentage of the *snt1*-*sz2* mutant cells exhibited highly condensed and “fragmented DNA” (Figure S3A,B). At G2/M, both the wild-type cells and the mutant cells displayed a single nucleus located in the mother cell, close to the neck. However, at later stages of mitosis, unlike in the wild-type cells, a high proportion of *stn1*-*sz2* mutant cells displayed a very faintly stained nucleus (if any nucleus was even present) and numerous pieces of condensed and fragmented DNA. The fragmented DNA localized all over the cells in both the mother and daughter compartments (Figure S3A).

To check whether the *stn1*-*sz2* “DNA fragmentation” phenotype was due to apoptosis, we genetically inactivated several apoptotic pathways in the *stn1*-*sz2* mutants [41]. Interestingly, the genetic inactivation of *AIF1*, *NUC1*, *YCA1*, *BXI1*, or *NMA1* did not lead to the suppression of this phenotype (Figure S3B). Since the mitotic spindle is destabilized in the mutant, this fragmented DNA might represent chromosomes or pieces of chromosomes spreading out because of the improper attachment of kinetochores (the structures that bind centromeres to microtubules).

**Figure 3:**
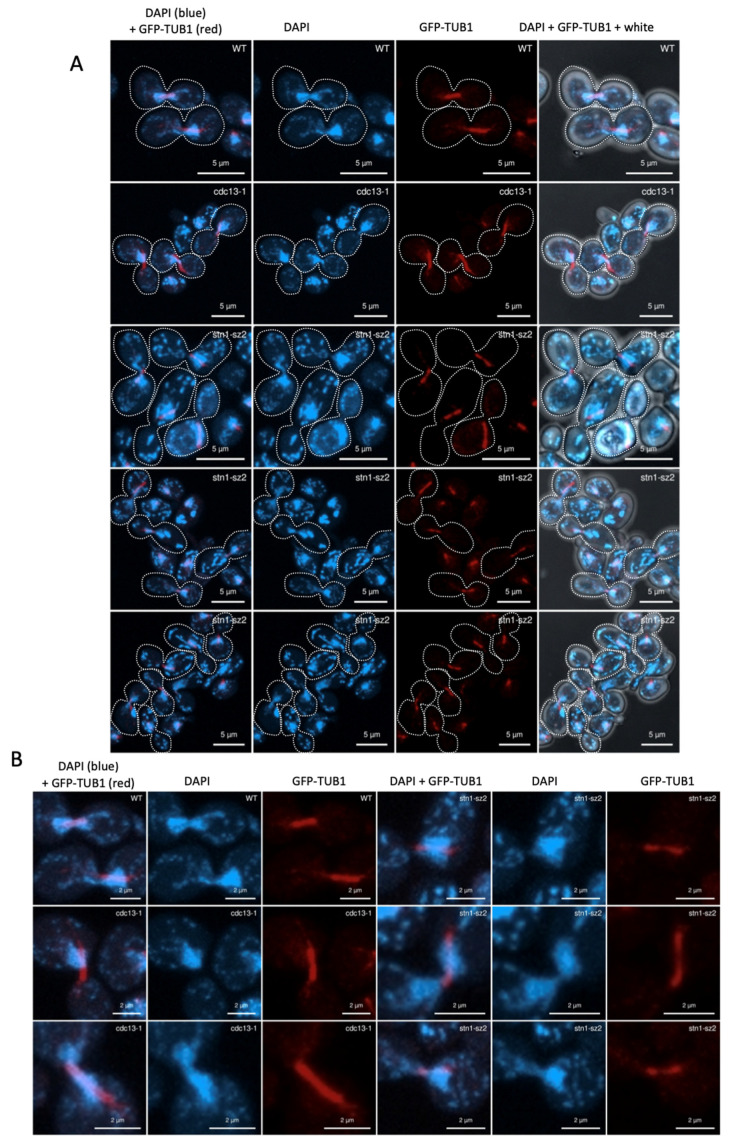
Comparison of the general organization of the mitotic spindle revealed by visualizing endogenous GFP-Tub1 (shown with red labeling) in *stn1*-*sz2*, *cdc13*-*1*, and wild-type cells, as indicated. DAPI staining was used to simultaneously visualize nucleus DNA (shown with blue labeling). (**A**) Cells were grown at 24 °C and then transferred to 36 °C for 2 h (at a restrictive temperature for *cdc13*-*1* and *stn1*-*sz2*). Cells were fixed with 37% formaldehyde for 10 min and then mounted on slides using the DAPI-containing Vectashield mounting medium. Pictures were taken using a Zeiss LSM 800 Airyscan confocal microscope. For all three strains, only metaphase cells were chosen for pictures. The GFP-Tub1 signal was represented in red, rather than green, for better visualization. (**B**) Higher magnifications of some of the photographs that are shown above in panel (**A**).

Next, we used an alternative protocol for GFP-Tub1 visualization, in which a 10 min formaldehyde treatment was used, thus avoiding the rapid depolymerization of tubulin during sample preparation. This alternative protocol was found to provide an optimal visualization of the mitotic spindle with GFP-Tub1 [42]. Indeed, without fixation, the GFP-Tub1 signal in the mitotic spindle was much less clear, as illustrated above (Figure S2). Under these conditions of formaldehyde fixation, G2/M cells exhibited a clearly visible mitotic spindle, extending across the nucleus (Figure 3). As explained in the section above, the *cdc13*-*1* mutant was also used (in addition to a wild type) as a control. In the *stn1*-*sz2* cells that had a normal nucleus (that is, those not exhibiting DNA fragmentation), the GFP-Tub1 signals were weaker and the mitotic spindles were thinner than in the wild-type cells (Figure 3). The *stn1*-*sz2* cells that exhibited a normal nucleus were the only ones that could be used in comparative quantitative analyses (Figure S4). Altogether, these experiments suggested that, in the *stn1*-*sz2* mutant, the stability of the mitotic spindle was profoundly altered.

### 3.3. The stn1-sz2 Mutant Exhibits Genetic Interactions with Mutants of STU1, STU2, TUB2, and SLK19

**Figure 4:**
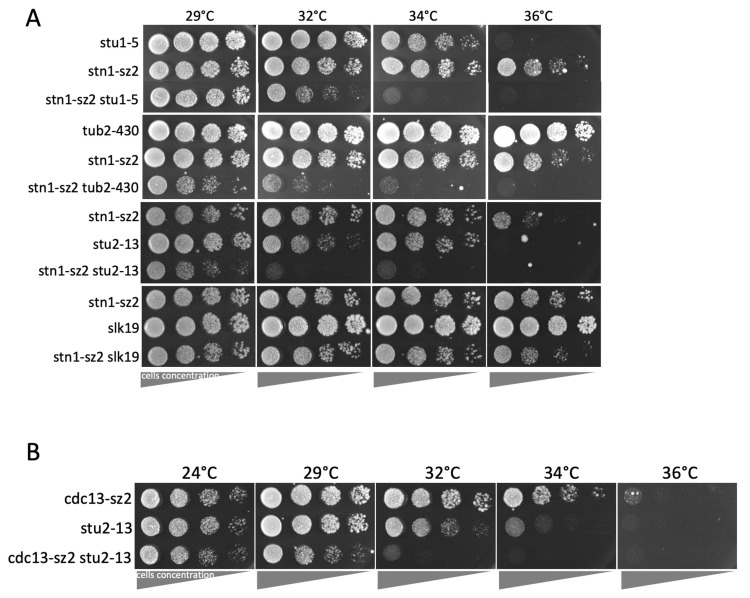
Genetic interactions between the *stn1*-*sz2* (**A**) or *cdc13*-*sz2* mutants (**B**) and mutants of either *STU1*, *STU2*, *TUB2*, or *SLK19*. “Spot assays” (or “drop tests”) were performed using tenfold serial dilutions (from left to right) of yeast cultures grown for 2 days on YEPD plates at the indicated temperatures.

The data above indicate that CST is greatly involved in the stability of the mitotic spindle. To find additional clues, we analyzed the genetic interactions between the *stn1*-*sz2* mutant and a fairly large number of mutants previously implicated in spindle functioning and/or integrity. The *stn1*-*sz2* mutation exhibited strong negative genetic interactions with the *stu1*-*5* mutation [43,44], the *stu2*-*13* mutation [45], and the *tub2*-*430*Δ mutation [46,47]. Moreover, we found that the complete deletion of the *SLK19* gene induced a discrete synthetic growth defect when combined with the *stn1*-*sz2* mutation. The corresponding data are illustrated in Figure 4A. The strong synthetic growth defects between *stn1*-*sz2* and *STU1*, *STU2*, and *TUB2* mutants discovered here suggest that Stn1 (and perhaps the entire CST complex) participates in spindle stability pathways normally controlled by Stu1, Stu2, and Tub2 in an independent but complementary manner. As a complement to these experiments, we found that neither the overexpression of *STU1* or *STU2* nor of *TUB2* or *TUB1* could rescue the temperature sensitivity of the *stn1*-*sz2* mutant (Grandin and Charbonneau, unpublished data). 

In order to establish whether these genetic interactions were specific to this particular *stn1*-*sz2* mutant or, rather, constituted a general characteristic of the CST complex, we analyzed the *cdc13*-*sz2 stu2*-*13* double mutant, with *cdc13*-*sz2* being a mutant of *CDC13* isolated in the same study in which *stn1*-*sz2* was isolated, sharing the characteristic of being rescued by *SIZ1* overexpression [28]. Interestingly, *cdc13*-*sz2* also exhibited a strong synthetic growth defect concerning *stu2*-*13* (Figure 4B).

### 3.4. Dynamics of the Spindle Pole Bodies in the stn1-sz2 Mutant

**Figure 5:**
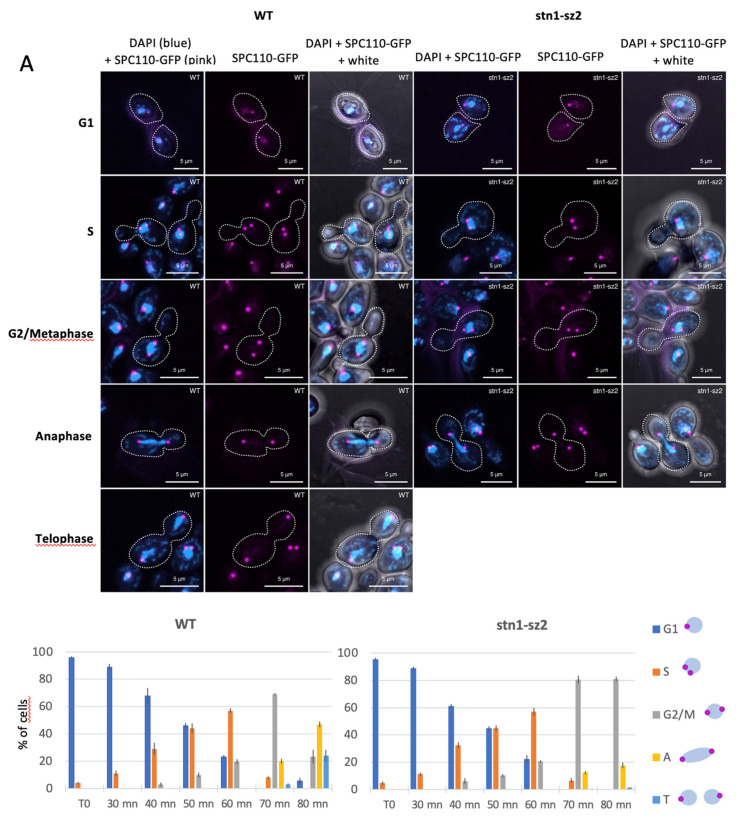

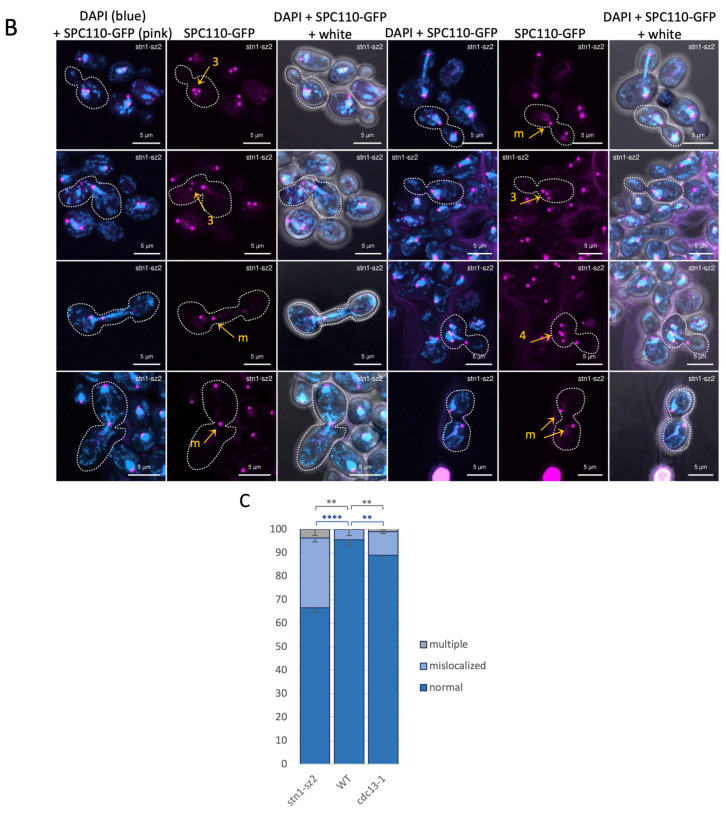
Localization and organization of the spindle pole bodies (SPBs) in *stn1*-*sz2* mutant cells. (**A**) Wild-type (WT) *bar1*Δ and *stn1*-*sz2 bar1*Δ were synchronized in G1 using alpha-factor and released at 36 °C before samples were taken at intervals to follow the SPBs’ dynamics using an *SPC110*-GFP integrated construct. Representative SPB localizations are shown in top panels. Three independent experiments were performed, and quantification is shown in bottom panels (histograms), in which G2/M represents G2/metaphase, A is anaphase, and T represents telophase. Cells with fragmented nuclei were not taken into account. (**B**) *stn1*-*sz2* mutant cells bearing an *SPC110*-GFP integrated construct were transferred to the restrictive temperature of 36 °C for 2 h. “m” stands for mislocalized, and “3” and “4” indicate the numbers of SPBs. In both (**A**,**B**), cells were mounted on slides using the DAPI-containing Vectashield mounting medium. Pictures were taken using a Zeiss LSM 800 Airyscan confocal microscope. Pink (instead of green) was used to better visualize the GFP signal. (**C**) Quantification of the mislocalized and multiple (i.e., more than the normal two SPBs) SPB signals in *stn1*-*sz2* cells after 2 h of growth at 36 °C. Results from 7 independent experiments for the WT and *stn1*-*sz2* and 4 independent experiments for *cdc13*-*1* are shown as percentages of total cells (with 100 cells counted per experiment). Significance numbers are given as *p*-values from the Student’s *t*-test (in blue for mislocalized SPBs and in gray for multiple SPBs). ** stands for a *p*-value < 0.01 and **** indicates a *p*-value < 0.0001.

The mitotic spindle is attached at each of its poles to the spindle pole bodies (SPBs), the homologs of the centrosomes in higher eukaryotes, whose main function is to nucleate the microtubules [48]. Since our findings highlighted the existence of a probable spindle defect in the *stn1*-*sz2* mutant, the dynamics of the SPBs during the cell cycle were examined next. To this end, we used strains harboring an *SPC110*-GFP construct integrated at the *SPC110* locus (Figure 5). Wild-type and *stn1*-*sz2* mutant cells were synchronized in G1 with alpha factor. Upon releasing the cells from the alpha factor block at 36 °C, samples were taken every 10 min to follow the SPBs’ dynamics (Figure 5A). The duplication and separation of the SPBs from G1 to early mitosis (G2/metaphase) occurred with the same kinetics in the wild-type and the mutant cells (cells with a fragmented nucleus were not taken into account for the reasons explained above). Noticeably, in mitosis, *stn1*-*sz2* mutant cells were clearly delayed, with most of the cells being in metaphase, while wild-type cells had already progressed through anaphase and telophase (Figure 5A). Moreover, it was also clear that a significant percentage of *stn1*-*sz2* cells displayed more than two SPBs or mislocalized SPBs, not aligned with the two poles of the DAPI-stained nucleus (Figure 5B,C). These additional data on the SPBs confirmed that the *stn1*-*sz2* mutation provoked mitotic spindle dysfunctions.

Using strains harboring *RFA1*-GFP (expressed from the *RFA1* genomic locus), we next set out to compare the number of nuclear Rfa1 loci between the *stn1*-*sz2* and *cdc13*-*1* mutants, specifically during anaphase (Figure S5). Rfa1 is known to be recruited to sites of DNA damage due to its high affinity with single-stranded DNA [49], and was therefore expected to form foci in the *cdc3*-*1* mutant, which is known to accumulate very high amounts of telomeric single-stranded DNA as a result of the loss of telomere end protection by Cdc13 [2]. Interestingly, both the *cdc13*-*1* and *stn1*-*sz2* mutants displayed high quantities of nuclear Rfa1 foci in anaphase cells and were not different from each other (Figure S5). Thus, the prototype tubulin-spindle *stn1*-*sz2* mutant shares with *cdc13*-*1* the characteristic of generating high amounts of single-stranded DNA. In addition, these experiments established that *stn1*-*sz2* mutant cells were enriched, in comparison with the *cdc13*-*1* mutant, in the proportion of cells in anaphase (Figure S5).

### 3.5. Dynamics of the Kinetochores in the stn1-sz2 Mutant

**Figure 6:**
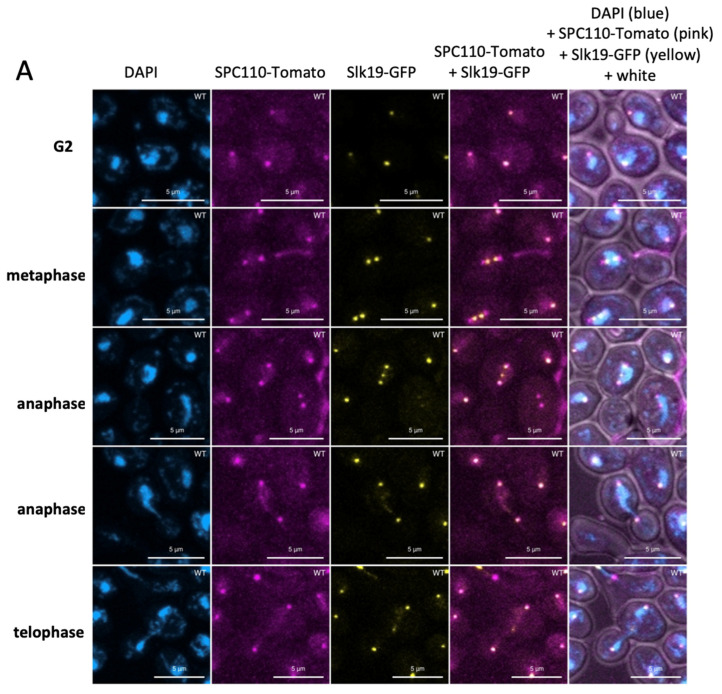

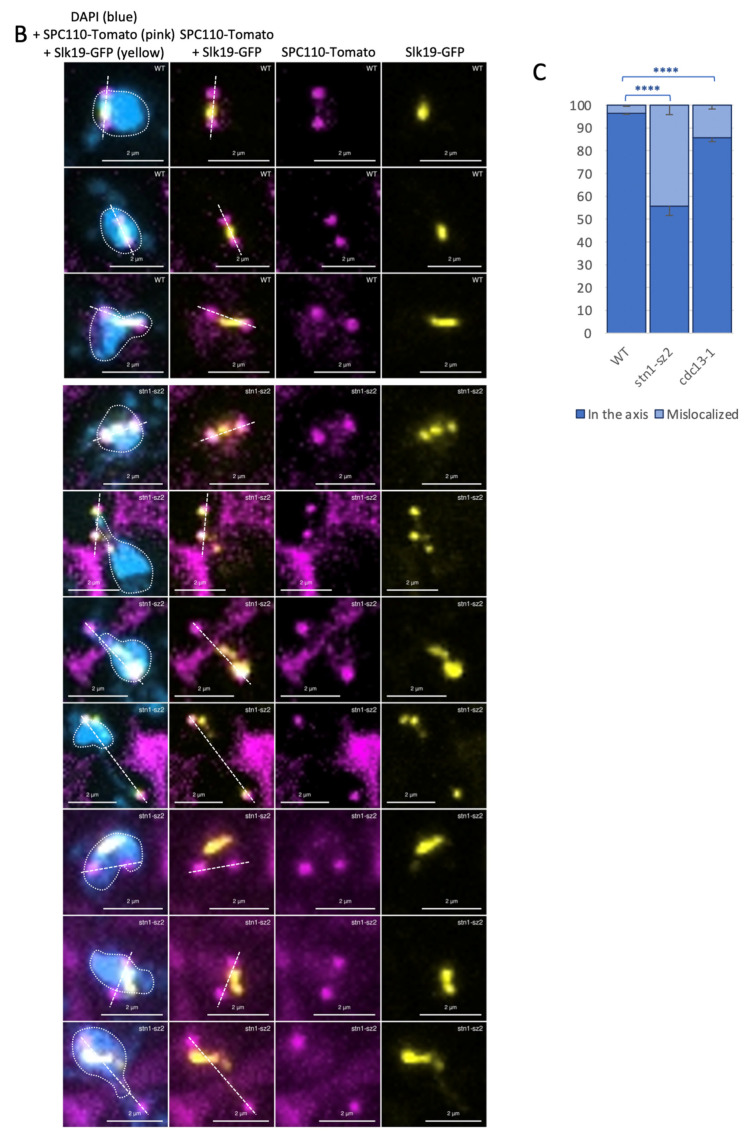
Localization and organization of the kinetochores in *stn1*-*sz2* mutant cells. (**A**) Wild-type (WT) cells bearing *SPC110*-tdTomato and *SLK19*-GFP integrated constructs were grown at 29 °C. Normal localization of the spindle pole bodies (SPBs) and kinetochores throughout mitosis (from G2 to telophase) are shown. (**B**) WT and *stn1*-*sz2* cells bearing the *SPC110*-Tomato and *SLK19*-GFP integrated constructs were grown at 24 °C and then transferred to 36 °C (a restrictive temperature for *stn1*-*sz2*) for 2 h. Only metaphase cells were used for pictures. This panel provides enlargements of the photographs presented in Figure S6. A dashed white line was drawn to indicate the axis between the two SPBs, corresponding to the normal localization of the metaphase spindle to which kinetochores should be attached. For both (**A**,**B**), cells were mounted on slides using the Vectashield with DAPI mounting medium. Pictures were taken using a Zeiss LSM 800 Airyscan confocal microscope. Pink (instead of red) and yellow (instead of green) were used to show the Tomato and GFP signals, respectively, to allow optimal visualization. (**C**) Quantification of the mislocalized kinetochore signals in the indicated strains’ cells after 2 h growth at 36 °C. In all three strains, only cells in metaphase were taken into account. Results from 4 independent experiments for each strain are given as percentages of total cells (with 100 cells counted per experiment). Significance numbers are *p*-values from the Student’s *t*-test (**** indicates a *p*-value < 0.0001).

Kinetochores are composed of a large multiprotein complex arranged around the centromeric structures of chromatids that attach to the kinetochore microtubules in order to keep the sister chromatids attached to the mitotic spindle until they can be correctly segregated at anaphase [48]. We set out to visualize kinetochore localization with respect to the spindle axis during mitosis. For this purpose, we used an *SLK19*-GFP construct together with an *SPC110*-tdTomato construct (Figure 6 and Figure S6). Slk19 is known to be localized to the kinetochores throughout mitosis. Slk19-labeled kinetochores were concentrated in foci in early mitosis and then pulled toward the SPBs early in metaphase, subsequently migrating with them towards the spindle poles during anaphase and telophase. We also observed a transient kinetochore-independent localization of Slk19 in the spindle midzone during anaphase (Figure 6A). Therefore, throughout mitosis, the Slk19-GFP signal should coincide with the mitotic spindle in the alignment of the two SPBs. It should be noted that Slk19 also localized with kinetochores not correctly attached to the spindle. Compared to wild-type cells, a high proportion of *stn1*-*sz2* cells displayed a mislocalized Slk19-GFP signal, suggesting a defect in the kinetochore’s attachment to the spindle (Figure 6B and Figure S6).

## 4. Discussion

### 4.1. stn1-sz2 Is the Prototype for a Mitotic-Spindle-Damage-Characterized cst Mutant

Here, we describe several phenotypes of *stn1*-*sz2*, a temperature-sensitive mutant of the budding yeast *S*. *cerevisiae*, with Stn1 being a subunit of the conserved telomeric Cdc13-Stn1-Ten1 complex, CST [5,12,13]. The *stn1*-*sz2* mutant, which was recently isolated in our laboratory, possesses previously unknown phenotypes, indicating a strong implication in the checkpoint pathways that detect damage to the tubulin mitotic spindle [28].

Throughout this study, we compared these new *stn1*-*sz2*′s phenotypes with those of *cdc13*-*1*, which is considered the prototype of the telomeric-DNA-damage-characterized *cst* mutants [2,39]. Most mutants of CST isolated to date exhibited a loss of protection of the telomeric DNA sequences, and the resulting accumulation of single-stranded DNA triggered the activation of the DNA damage checkpoint (DDC) [50]. We have found phenotypes present in the *stn1*-*sz2* mutant but not in the *cdc13*-*1* mutant, and these are related to the stability of the tubulin mitotic spindle (see below). Thus, we propose to define *stn1*-*sz2* as the prototype of mitotic-spindle-damage-characterized *cst* mutants, providing a useful tool with which to elucidate potential new CST functions. 

The analysis of *stn1*-*sz2* mutants harboring mutations in two of the three major mitotic checkpoints (Figure 2A) established that the strong synthetic lethality affecting the *stn1*-*sz2 bub2*Δ double mutant was ameliorated when either *MAD1* or *RAD17* was deleted. The most likely interpretation of these events is as follows: the delay in cell cycle progression induced by the SAC or the DDC provoked some damage that was further recognized by the SPOC. Indeed, when the SAC or the DDC were inactivated in the *stn1*-*sz2 bub2*Δ double mutant, the arrest normally recognized by the SPOC and resulting in death no longer occurred (Figure 2B,C). We propose that the damaged spindle structure that is normally recognized by the SPOC is no longer present in *mad2*Δ or *rad17*Δ cells because the cell cycle has progressed beyond the state that activates the SPOC. These data suggest that the SAC and the SPOC may not be simultaneously activated, with the SPOC being activated only if both the DDC and the SAC are also activated.

Given that *stn1*-*sz2* is more sensitive to the inactivation of the SAC and the SPOC than *cdc13*-*1*, it is possible that the *stn1*-*sz2*-induced cell cycle arrest takes place later than the *cdc13*-*1* arrest. In other words, *stn1*-*sz2*-induced DDC arrest might be leaky, allowing the *stn1*-*sz2* mutant cells to progress further into the cell cycle than the *cdc13*-*1* mutant cells. This interpretation might explain why the *cdc13*-*1* mutation did not activate the SAC [34].

### 4.2. A Major Defect of stn1-sz2 Is the Stability of the Mitotic Tubulin Spindle

The existence of functional interactions between *stn1*-*sz2* and both major G2/M spindle checkpoints, as discussed above, prompted us to investigate the status of the tubulin mitotic spindle. Our data demonstrate the existence of a profound instability of the mitotic spindle in the *stn1*-*sz2* mutant in comparison with either the wild type or the *cdc13*-*1* mutant (Figure 3 and Figure S4). Proper chromosome segregation in mitosis is controlled by dynamic interactions between the spindle microtubules and the kinetochores, as well as the position of the SPBs (spindle pole bodies or centrosomes) during spindle elongation [48,51]. Accordingly, we concentrated on describing new *stn1*-*sz2* phenotypes associated with these structures and found profound defects in the organization of the SPBs (Figure 5) and kinetochores (Figure 6 and Figure S6). Whether these defects are provoked by tubulin defects or just accompany them—or, rather, provoke them—is not yet known. Interestingly, a small candidate genetic screen identified strong genetic interactions of the *stn1*-*sz2* mutant with *stu1* and *stu2* mutants as well as with the *tub2*-*430*Δ mutant.

Tub2 (beta-tubulin) associates with alpha-tubulin (Tub1 and Tub3) to form tubulin dimers, which polymerize to form microtubules [52]. *STU1* was isolated as a suppressor of a *tub2* mutant [43], and the Stu1 protein physically associates with Tub2, being required for the integrity of the mitotic spindle [44]. Stu1 is a microtubule plus-end-tracking non-motor protein that stabilizes interpolar microtubules’ plus ends, facilitating the polymerization of spindle microtubules and exerting an outward force on spindle poles [44]. In addition, Stu1 binds to detached kinetochores and promotes their capture, stabilizing the spindle once the captured kinetochores reach a spindle pole [53,54]. Interestingly, Slk19 localizes both to the midzone (like Stu1) in anaphase and the kinetochores throughout mitosis [55,56]. In addition, Slk19′s localization to the metaphase spindles was recently found to be dependent on Stu1 [57]. Stu2, on the other hand, is a microtubule polymerase that promotes microtubule elongation through interactions with alpha–beta-tubulin heterodimers [58]. Stu2 has multiple functions at the kinetochore, particularly in the attachment of the kinetochore microtubules and the selection of correctly oriented kinetochore attachments and spindle orientation [45,59,60].

During the course of the present study, we became interested in the interactions between tubulin and CST after observing the dramatic disorganization of the tubulin spindle in the *stn1*-*sz2* mutant. Then, we became particularly interested in a mutant of tubulin lacking the β-CTT (Carboxy Terminal Tail), referred to as the *tub2*-*430*Δ mutant, because, unlike α-CTT mutants, it was found to be defective in spindle stabilization and chromosome segregation [46,47]. In normal cells, the sister chromatids must not segregate to the spindle poles until there is no obstacle to their separation up to their very ends, that is, the telomeres. If there is some telomeric damage, as with the *stn1*-*sz2* mutant in this study, the mitotic spindle must be stabilized until the telomeres are free of damage and can therefore be correctly segregated; otherwise, the whole spindle breaks down. We propose that the “late function” of the CST complex identified here might be involved in the stabilization of the mitotic spindle. In the *stn1*-*sz2* mutant, this spindle stabilization is compromised, hence the very dramatic associated phenotypes described here. There might be particular events devoted to specific physical interactions, or other types of interactions, between CST (or perhaps just Stn1) and some structure/pathway found here to be strongly affected by the *stn1*-*sz2* mutation. The identification of *stu1* and *stu2* mutants, as well as of the *tub2*-*430*Δ mutant, exhibiting negative genetic interactions with *stn1*-*sz2*, strongly suggests that Stu1 and Stu2 play an important role in relaying information from CST to the tubulin spindle. However, there are not enough data to propose a model.

The current data provide completely new information on the functions of the CST telomeric complex. As a result, many gaps remain to be filled, and the most plausible explanation (in our opinion) described in the previous paragraph is not the only possible one. Indeed, it is not always possible to distinguish causal from correlative molecular events. For example, the damage generated by *stn1*-*sz2* could affect a mechanism or pathway occurring upstream of an execution point required to initiate spindle assembly mechanisms. In this case, the spindle organization defect observed in the *stn1*-*sz2* mutant would represent a parallel outcome, not a direct downstream consequence. Identifying the molecular events that allow the control of spindle organization by CST, by performing experiments that challenge the elements of the model presented in the previous paragraph, should help resolve these issues.

At this stage of the research project, it might be interesting to determine, using specific assays, whether the *stn1*-*sz2* mutant actually exhibits telomere cohesion defects. The most advantageous method to achieve this would be to analyze these parameters concomitantly using live-cell imaging. Furthermore, the current difficulties in identifying the precise interactions between CST and the spindle regulators involved, namely Stu1, Stu2, and tubulin, could be overcome by performing new genetic screens aimed at identifying the missing links between these proteins. Ideally, these genetic screens could identify conditional mutants of *STU1*, *STU2*, or *TUB2* whose growth could be improved by *STN1* overexpression. Finally, it will be important to understand the role of Siz1, a SUMO E3 ligase, in the involvement of CST in spindle organization, since the *stn1*-*sz2* mutant was initially isolated on the basis of being rescued by *SIZ1* overexpression [28]. Given the close physical interactions between Siz1 and septins, on the one hand, and the close genetic interactions between septins and CST, on the other hand, we speculate that Siz1 could be a target of *stn1*-*sz2* damage-activated checkpoints, acting to control mitotic exit in cooperation with septins.

To the best of our knowledge, the data reported herein are the first to describe a mutant of CST exhibiting a phenotype predominantly associated with defects in the mitotic spindle. On the other hand, previous studies on mice and humans uncovered a co-localization and physical interaction between TRF1, a subunit of the shelterin telomeric complex, and the SAC proteins MAD1, MAD2, and BUBR1 [61,62]. More recently, human TRF1 was found to ensure proper chromosome segregation by regulating Aurora-B’s centromeric function required for microtubule–kinetochore attachment [63]. Intriguingly, the chromosome segregation abnormalities induced by TRF1 depletion in these cells were independent of the telomeric function of the protein [63].

### 4.3. The stn1-sz2 Mutant Exhibits “DNA Fragmentation”

Incidentally, an additional interesting phenotype was found in the *stn1*-*sz2* mutant, namely, a very dramatic fragmentation of the nuclear DNA. We suggest that this fragmented DNA might represent chromosomes or pieces of chromosomes spreading because of kinetochores that do not properly attach to the mitotic spindle, possibly because the mitotic spindle is destabilized. It is tempting to assume that the fragmented DNA we show here represents structures observed in mammalians and humans known as micronuclei [64], but we cannot be certain that this is the case. The accumulation of extranuclear micronuclei is a hallmark of cancer, inflammatory-associated diseases, and aging. Interestingly, the presence of micronuclei is associated with situations in which chromosome segregation during anaphase is perturbed by lagging acentric chromatids (for instance, [65]), and, in this respect, it might be worth continuing to characterize these structures in the *stn1*-*sz2* mutant.

### 4.4. Implication of the New stn1-sz2 Phenotypes for Cancer Research

Mutations in human CTC1 and STN1 have mainly been associated with *dyskeratosis congenita* and Coats Plus ([24,26] and references therein). In addition, STN1 downregulation and/or mutations have been reported in a number of cancers, including colorectal cancer, epithelial ovarian cancer, leukemia, thyroid cancer, melanoma, and uterine cancer ([25,26] and references therein). Given the phenotypes described here concerning the budding yeast *stn1*-*sz2* mutant, these pathologies might stem not only from deregulation in CTC1-STN1-Pol-α-telomerase interactions [66] but also potential defects in mitotic spindle stability.

It is very likely that the CST-controlled pathways described here in budding yeast have been conserved during evolution and also exist in humans. The new findings of the present study will certainly allow the apprehension of new targets that are deregulated in cancer cells and the design of new approaches to blocking their proliferation.

## Data Availability

All the data supporting the reported results are present in this article (in the main text and Supplementary Materials). Additional data or protocol details can be made available upon request.

## Supplementary Materials

The following supporting information can be downloaded at https://www.mdpi.com/article/10.3390/cells14110784/s1, Figure S1: Impact of mitotic checkpoint inactivation on the growth of CST mutants. Figure S2: Mitotic spindle stability is severely affected in the *stn1*-*sz2* mutant. Figure S3: Nuclear “DNA fragmentation” phenotype in the *stn1*-*sz2* mutant. Figure S4: Visualization of mitotic spindles in *stn1*-*sz2*, *cdc13*-*1*, and wild-type cells, revealed by endogenous GFP-Tub1 (red labeling). Figure S5: Comparison of Rfa1 DNA damage loci in *stn1*-*sz2* and *cdc13*-*1* mutants harboring an endogenous *RFA1*-GFP construct. Figure S6: Kinetochore localization and organization in *stn1*-*sz2* mutant and wild-type (WT) cells carrying the integrated *SPC110*-tdTomato and *SLK19*-GFP constructs.
