# Supplementary material for: The stn1-sz2 Mutant Provides New Insight into the Impacts of Telomeric Cdc13-Stn1-Ten1 Dysfunction on Cell Cycle Progression
Source: Cells. 2025 May 26;14(11):784. doi: 10.3390/cells14110784 (PMC12153855; doi:10.3390/cells14110784)
Supplement: Supplementary file 1 [file cells-14-00784-s001.zip › cells-3593838-supplementary.pdf]

Figure S1

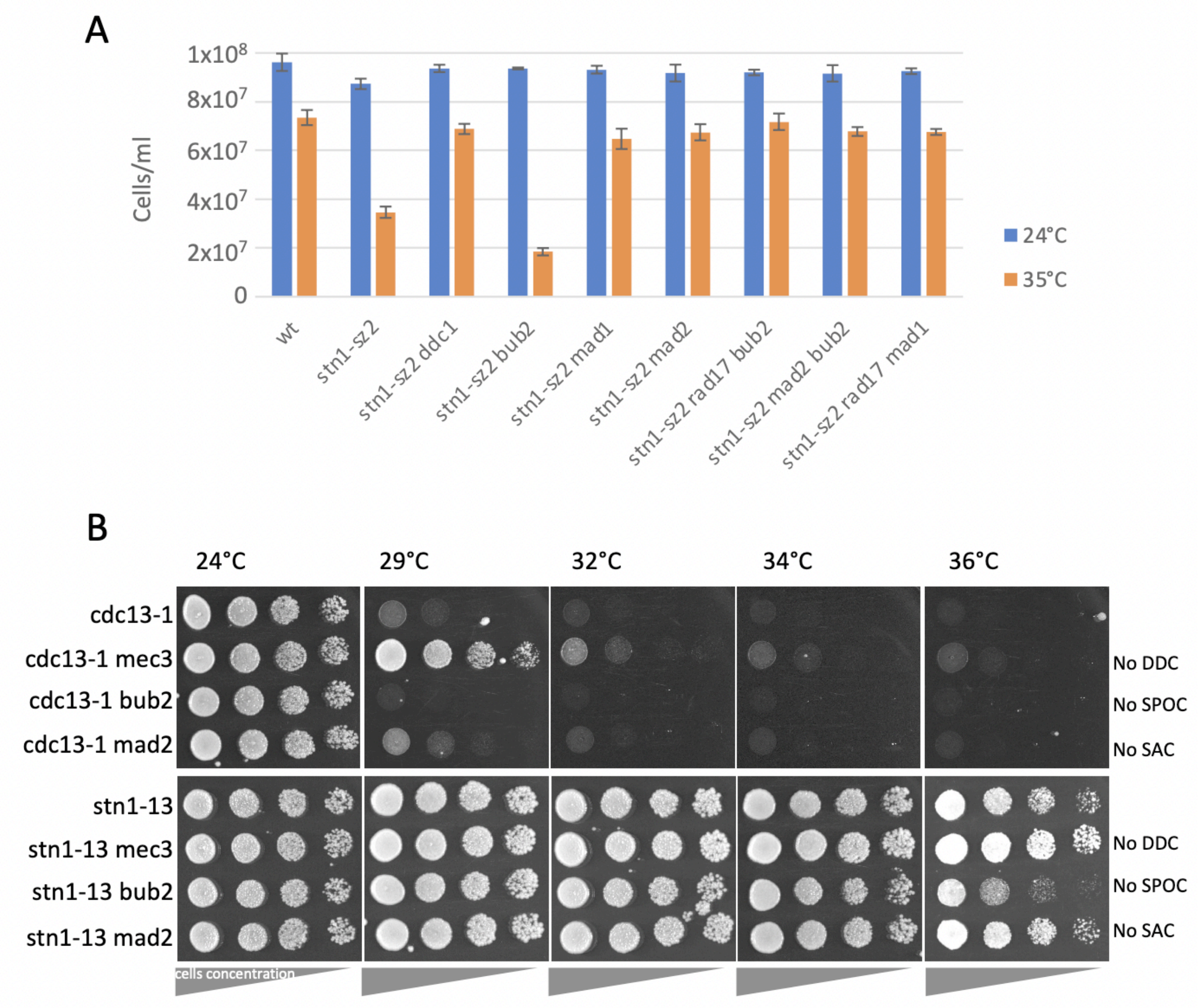

Figure S1. Impact of mitotic checkpoint inactivation on the growth of CST mutants. (**A**) Cell population growth measurements at 24°C (blue histograms) or 35°C (orange histograms). Liquid cultures of the indicated strains were grown at 24°C overnight, then diluted to 5 x 10^5 cells per ml and allowed to grow at 24°C or 35°C for 30 hr. Cells were then counted under a microscope. Results are from five independent experiments. (**B**) The *cdc13-1* and *stn1-13* mutations activate the DNA damage checkpoint (DDC). The *mec3*Δ mutation completely inactivates the DDC, while *bub2*Δ and *mad2*Δ completely inactivate the SPOC (Spindle Orientation Checkpoint) and SAC (Spindle Assembly Checkpoint), respectively. Serial dilutions of liquid cultures were spotted onto YEPD plates and grown at the indicated temperatures for 2 days. The growth defect generated by *cdc13-1* was highly sensitive to DDC inactivation, being only slightly aggravated by SPOC inactivation and very slightly ameliorated by SAC inactivation. In contrast, the growth defect generated by *stn1-13* was sensitive to both DDC and SPOC inactivation.

Figure S2

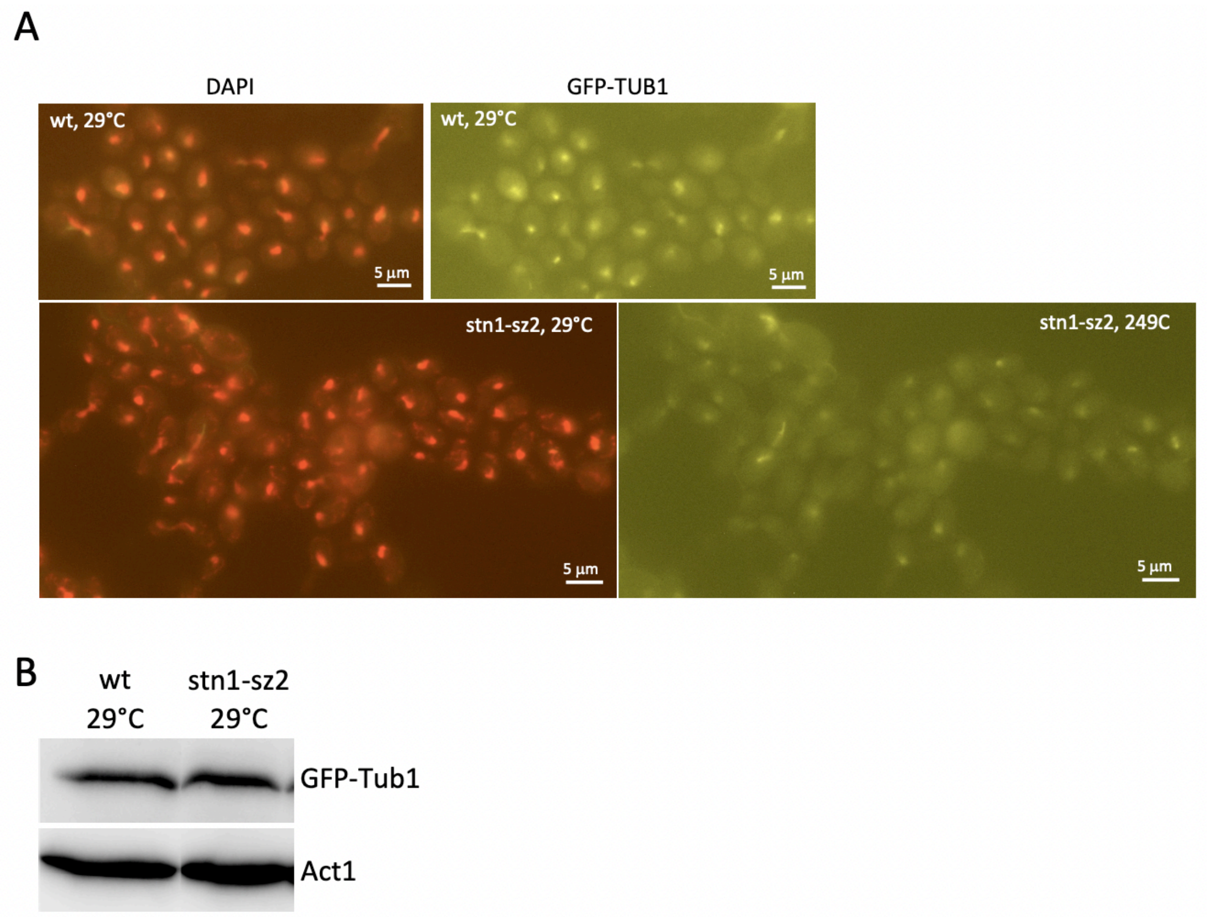

Figure S2. Mitotic spindle stability is severely affected in the *stn1-sz2* mutant. (**A)** Wild-type (wt) and *stn1-sz2* cells harboring an integrated GFP-*TUB1* construct were cultured at 29°C (permissive temperature for *stn1-sz2*). While a bright GFP-Tub1 signal was clearly observed in wild-type cells, in contrast, only a very faint signal, or even no signal, was visualized in *stn1-sz2* cells. The *stn1-sz2* mitotic spindles were also much thinner than those of the wild type. Cells were mounted on slides using Vectashield mounting medium with DAPI. Pictures were taken using a Zeiss fluorescence microscope. Red (instead of blue) and yellow (instead of green) were used to show the DAPI and GFP signals, respectively, for better visualization. (**B**) Western blotting of protein extracts from the same strains grown under the same conditions excluded the possibility that GFP-TUB1 was not properly expressed in *stn1-sz2* cells.

Figure S3

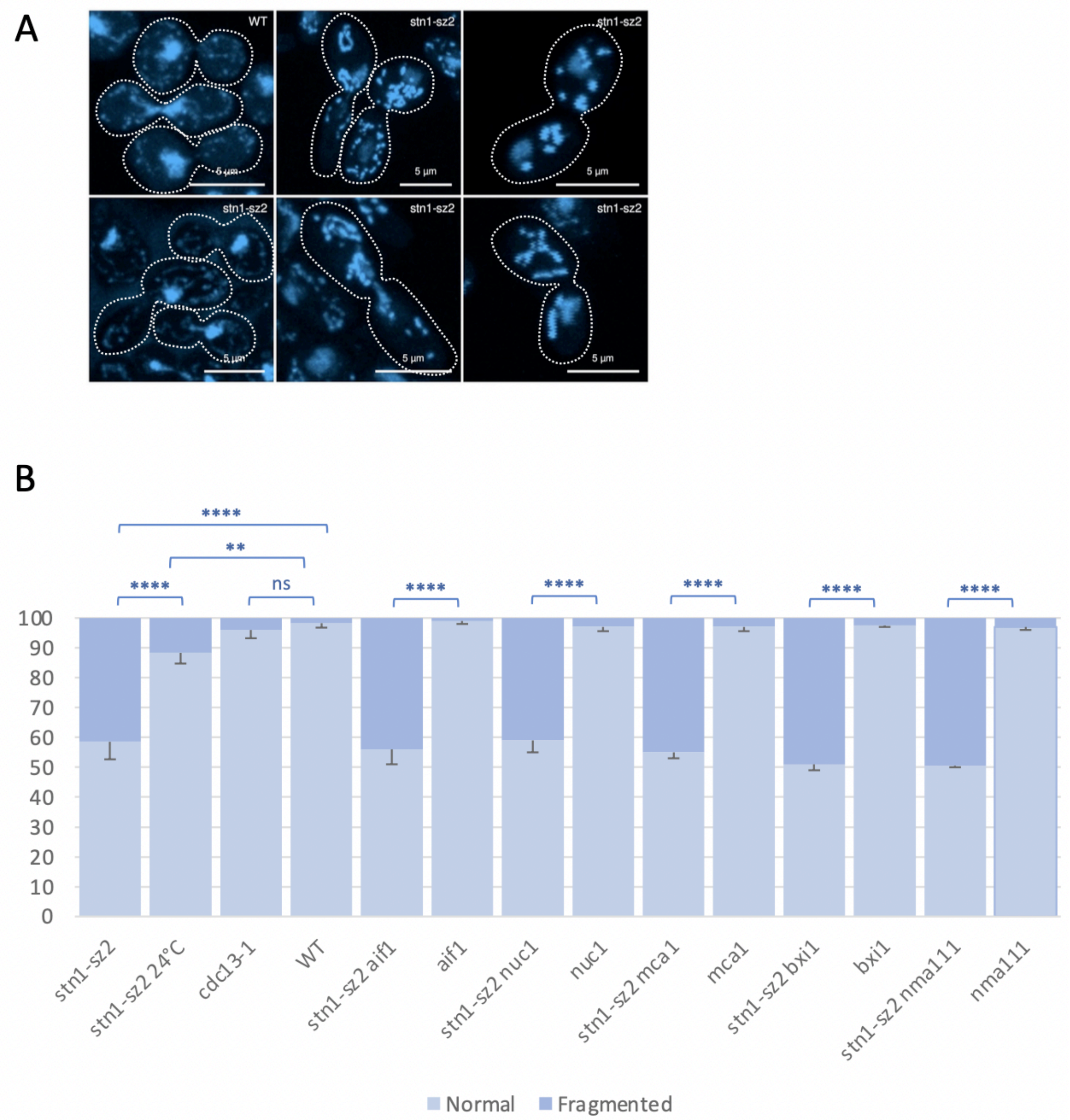

Figure S3. Nuclear "DNA fragmentation" phenotype in the *stn1-sz2* mutant. (**A**) After 4 hr at 34°C, a restrictive temperature for *stn1-sz2* at which mutant cells were delayed during the G2/M phase, both wild-type (top left) and mutant (bottom left) cells displayed a single nucleus located in the mother cell, near the neck between the mother and daughter cells. However, at later stages of mitosis, in contrast to wild-type cells (not shown), a high proportion of *stn1-sz2* mutant cells displayed a very weakly stained or even absent nucleus and numerous fragments of condensed and fragmented DNA (middle and right). Fragmented DNA was localized throughout the cell, in both the mother and daughter compartments. Cells were stained with DAPI, mounted in Vectashield medium, and observed under a Zeiss LSM 800 Airyscan confocal microscope. (**B**) Quantification of the presence of fragmented nuclei in the indicated strains, each carrying a mutation in a pathway essential for apoptosis. Cells were grown at 24°C in liquid medium and then transferred to 34°C for 4 hr (except for the indicated *stn1-sz2* strain, grown at 24°C). Results from at least 3 independent experiments are presented as a percentage of the total cell number. Significance is indicated by p-values from the Student's *t* -test.

Figure S4

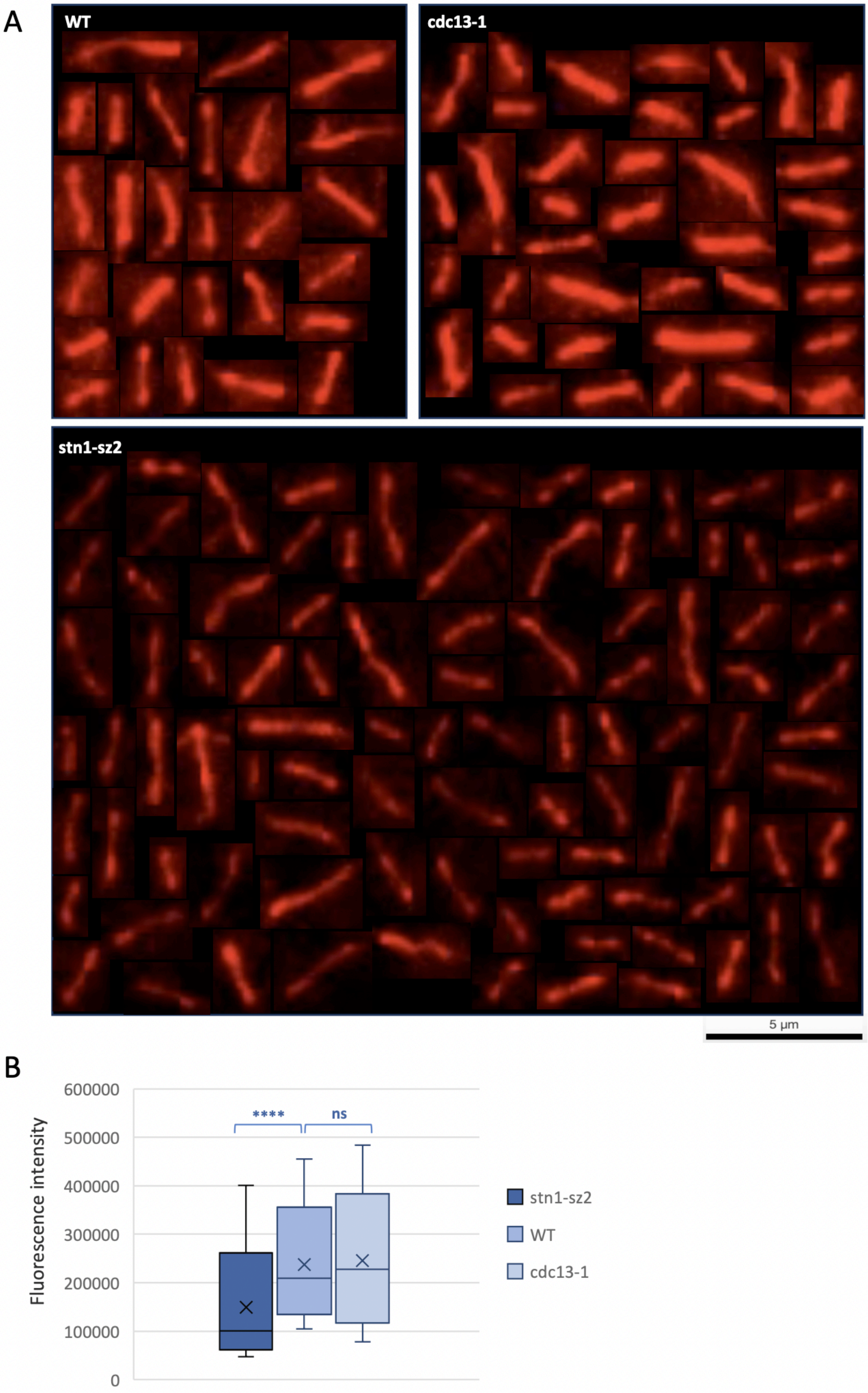
A
WT
cdc13-1
stn1-sz2
5 µm
B
Fluorescence intensity
600000
500000
400000
300000
200000
100000
0
****
ns
stn1-sz2
WT
cdc13-1

Figure S4. Visualization of mitotic spindles in *stn1-sz2*, *cdc13-1*, and wild-type cells, revealed by endogenous GFP-Tub1 (red labeling). (**A**) Metaphase spindles after transfer of the cell population to 36°C for 2 hr (at this temperature, most *cdc13-1* and *stn1-sz2* cells are arrested in G2/M). Pictures were taken using a Zeiss LSM 800 Airyscan confocal microscope. In all three cell populations, only metaphase spindles were considered. For better visualization, GFP signals were shown in red (instead of green). (**B**) Metaphase spindle intensities were quantified using Fiji software on photographs analyzed with Omero software. Quantification was performed on 165 *stn1-sz2*, 77 *cdc13-1*, and 59 WT metaphase spindles. Significance (p-values) was calculated using Student's *t*-test.

Figure S5

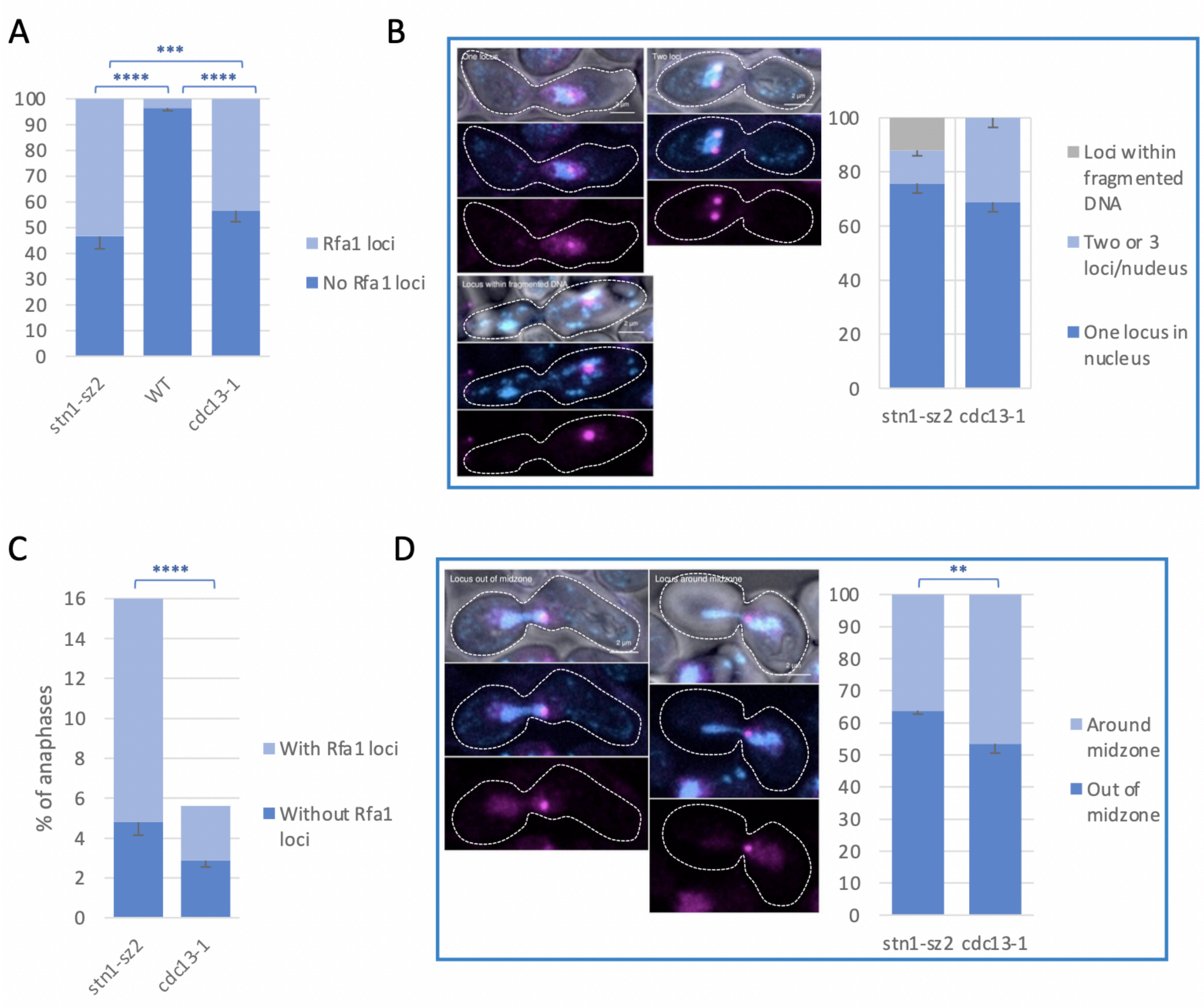

Figure S5. Comparison of Rfa1 DNA damage loci in *stn1-sz2* and *cdc13-1* mutants harboring an endogenous *RFA1*-GFP construct. Cells were grown at 24°C and then transferred to 36°C for 2 hr.

Cells were then mounted on slides using Vectashield mounting medium with DAPI and pictures were taken on a Zeiss LSM 800 Airyscan confocal microscope. Significance (p-values) was calculated using Student's *t*-test. (**A**) Percentage of nuclear Rfa1-GFP loci in the indicated strains. Results correspond to the mean values of 9 independent experiments each for the wild-type (WT) and *cdc13-1* strains and 11 independent experiments for the *stn1-sz2* strain (100 cells were counted in each experiment). (**B)** Number of Rfa1-GFP loci per cell for the indicated strains. Results are expressed as a percentage of the total number of cells showing a GFP signal. Five independent experiments for each strain (100 nuclei counted per experiment). Photographs illustrate typical Rfa1-GFP signals for each category: one locus per nucleus, multiple loci per nucleus (usually two), and GFP signals localized in fragmented DNA. DAPI in blue, GFP signals in pink. (**C**) Percentage of anaphase (elongated) nuclei in the neck between mother and daughter cells, showing or not showing Rfa1-GFP signals, in the indicated strains. In strain *cdc13-1*, cells that were not arrested in anaphase were arrested in G2/M with a round nucleus near the neck between mother and daughter cells. In the *stn1-sz2* strain, cells that were not arrested in anaphase were arrested in G2/M or had fragmented DNA (see Figure S3 for details). Results are from 3 independent experiments for each strain (500 cells counted per experiment). The *stn1-sz2* strain had significantly higher proportions of anaphase than the *cdc13-1* strain. (**D**) Localization of Rfa1-GFP signals in anaphase nuclei counted in (C) for the indicated strains. Photographs illustrate typical Rfa1-GFP signals for each category: signal close to the middle of the elongated nucleus (midzone of the mitotic spindle) or not. DAPI in blue, GFP in pink.

Figure S6

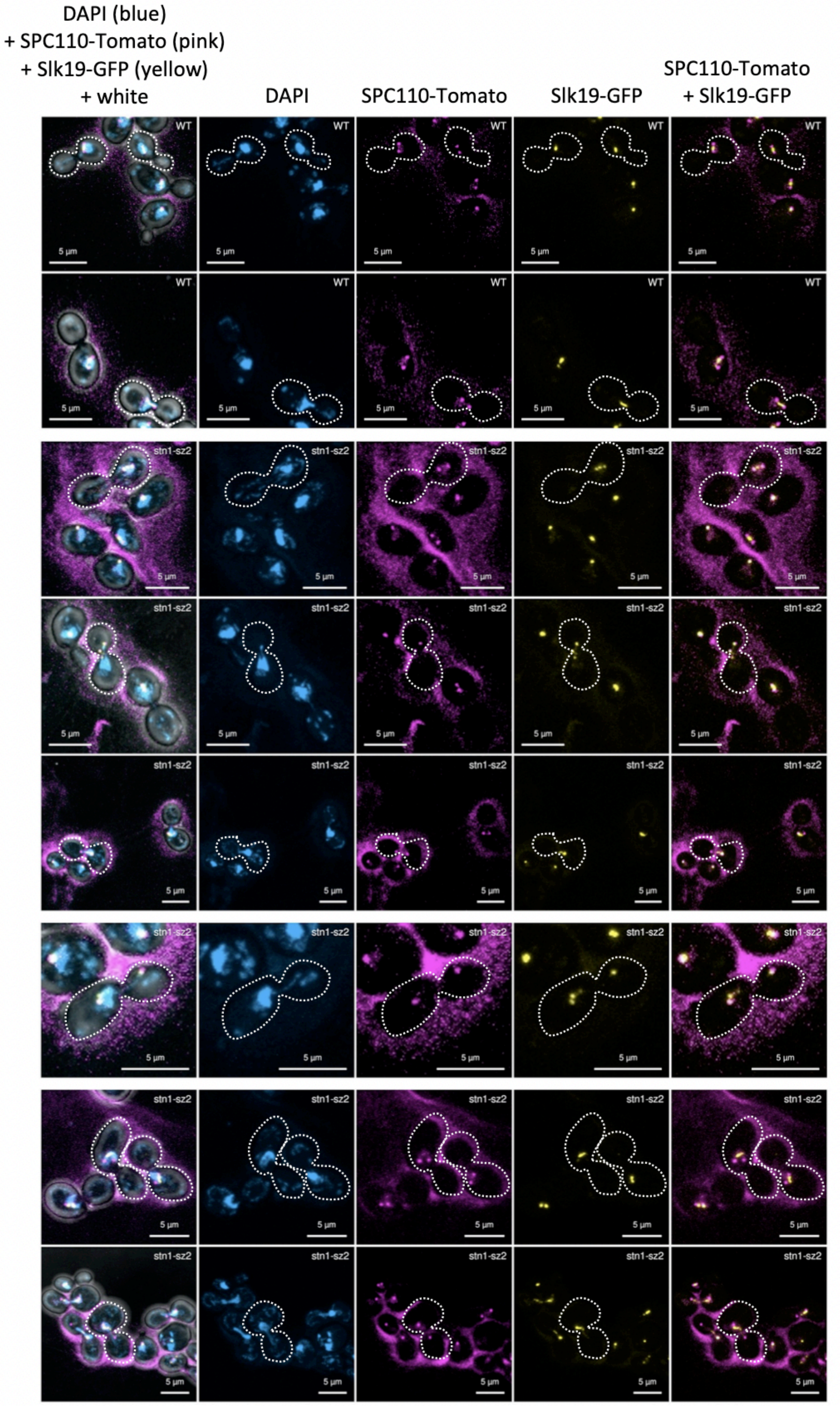

DAPI (blue)
+ SPC110-Tomato (pink)
+ Slk19-GFP (yellow)
+ white
DAPI
SPC110-Tomato
Slk19-GFP
SPC110-Tomato
+ Slk19-GFP
WT
stn1-sz2
5 μm

Figure S6. Kinetochore localization and organization in *stn1*-*sz2* mutant and wild-type (WT) cells carrying the integrated *SPC110*-tdTomato and *SLK19*-GFP constructs. Cells were grown at 24°C and then transferred to 36°C for 2 hr (restrictive temperature for *stn1*-*sz2*). Cells were then mounted on slides using Vectashield mounting medium with DAPI. Photographs were taken using a Zeiss LSM 800 Airyscan confocal microscope. Pink (instead of red) and yellow (instead of green) were used to represent Tomato and GFP signals, respectively, for better visualization.
